# A Single Dose of Neuron-Binding Human Monoclonal Antibody Improves Spontaneous Activity in a Murine Model of Demyelination

**DOI:** 10.1371/journal.pone.0026001

**Published:** 2011-10-12

**Authors:** Aleksandar Denic, Slobodan I. Macura, Arthur E. Warrington, Istvan Pirko, Brandon R. Grossardt, Larry R. Pease, Moses Rodriguez

**Affiliations:** 1 Department of Neurology, Mayo Clinic, Rochester, Minnesota, United States of America; 2 Department of Biochemistry, Mayo Clinic, Rochester, Minnesota, United States of America; 3 Department of Biomedical Statistics, Mayo Clinic, Rochester, Minnesota, United States of America; 4 Department of Immunology, Mayo Clinic, Rochester, Minnesota, United States of America; Innsbruck Medical University, Austria

## Abstract

Our laboratory demonstrated that a natural human serum antibody, sHIgM12, binds to neurons *in vitro* and promotes neurite outgrowth. We generated a recombinant form, rHIgM12, with identical properties. Intracerebral infection with Theiler's Murine Encephalomyelitis Virus (TMEV) of susceptible mouse strains results in chronic demyelinating disease with progressive axonal loss and neurologic dysfunction similar to progressive forms of multiple sclerosis. To study the effects of rHIgM12 on the motor function of TMEV-infected mice, we monitored spontaneous nocturnal activity over many weeks. Nocturnal behavior is a sensitive measure of rodent neurologic function because maximal activity changes are expected to occur during the normally active night time monitoring period. Mice were placed in activity boxes eight days prior to treatment to collect baseline spontaneous activity. After treatment, activity in each group was continuously recorded over 8 weeks. We chose a long 8-week monitoring period for two reasons: (1) we previously demonstrated that IgM induced remyelination is present by 5 weeks post treatment, and (2) TMEV-induced demyelinating disease in this strain progresses very slowly. Due to the long observation periods and large data sets, differences among treatment groups may be difficult to appreciate studying the original unfiltered recordings. To clearly delineate changes in the highly fluctuating original data we applied three different methods: (1) binning, (2) application of Gaussian low-pass filters (GF) and (3) polynomial fitting. Using each of the three methods we showed that compared to control IgM and saline, early treatment with rHIgM12 induced improvement in both horizontal and vertical motor function, whereas later treatment improved only horizontal activity. rHIgM12 did not alter activity of normal, uninfected mice. This study supports the hypothesis that treatment with a neuron-binding IgM not only protects neurons *in vitro*, but also influences functional motor improvement.

## Introduction

**Figure 1:**
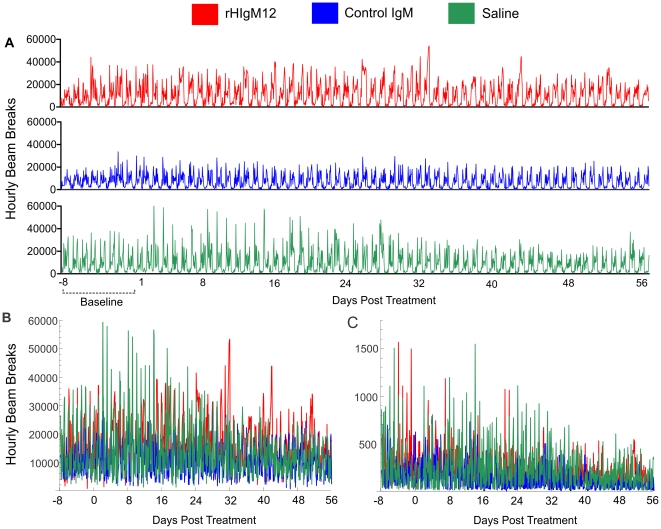
Example of spontaneous nocturnal activities of TMEV infected mice. **A)** Original complete recordings of horizontal activity over the period of 64 days. High nocturnal and low diurnal activities are easy to appreciate. Because mice are normally active during the night time we selected nocturnal activities for analysis (6PM-6AM). However, by visual inspection of unfiltered compressed recordings in either horizontal (B) or vertical (C) activity it may be difficult to identify real long-term changes.

Long-term monitoring and analysis of neurological function in rodent disease models remains a challenge. Nocturnal behavior is a sensitive measure of rodent neurologic function because maximal activity changes are expected to occur during the normally active night time monitoring period [1]. However, in animal models of slowly progressive diseases performance monitoring often extends over several weeks. We previously reported that oligodendrocyte-binding antibody (rHIgM22) enhanced remyelination by 5 weeks after the treatment [2]. Taken together that both the evolution of disease and repair is a slow process and to assure that any fluctuation in activity is taken into account, we monitored prolonged activity with the same sampling density used in short-term monitoring. This created large and highly fluctuating data sets (Figure 1A-C). To clearly delineate post-treatment changes and recover general trends in long-term activity, we compared the use of data binning, Gaussian filtering, and polynomial fitting by using Mathematica (Wolfram Research, Inc.).

Intracerebral infection with Theiler's Murine Encephalomyelitis Virus (TMEV) of susceptible mouse strains results in chronic demyelinating disease with progressive neurologic dysfunction similar to progressive forms of multiple sclerosis [3]. Treating this model with IgM class antibodies that bind to oligodendrocytes improves CNS remyelination [4]. In contrast, a serum-derived human monoclonal antibody (sHIgM12) that binds to neurons, promotes robust neurite outgrowth to the same degree as laminin, and reduces the inhibitory effects of CNS myelin on neurite outgrowth [5]. More recently a recombinant form of human sHIgM12 was generated (rHIgM12) with identical biological properties. We showed previously that rHIgM12 has a half-life of 3.6 hours, but still crosses the blood brain barrier and binds to nervous tissues (unpublished observation). To study the effects of rHIgM12 on the activity of TMEV-infected mice we monitored spontaneous activity over several weeks using AccuScan activity boxes (Accuscan Instruments, Inc., Columbus, OH). We chose a relatively long 8-week monitoring period for two reasons: (1) IgM induced remyelination is present by 5 weeks post treatment, and (2) TMEV-induced demyelinating disease in this strain progresses very slowly compared to the purely autoimmune EAE models of MS [6]. Due to the long observation periods compared to previously published studies (Table 1), identifying changes by visual inspection of raw data proved difficult.

**Table 1:** Length of the spontaneous activity monitoring in published studies compared to current study.

Publication	Length of recorded locomotor activity session
Mikami et al. 2002, 2004 [14], [15]	10 minutes (10 minutes of habituation)
Melnick and Dow-Edwards 2001 [16]	60 minutes (no habituation)
Chen et al. 2005 [17]	30 minutes (no habituation)
Torres-Reveron and Dow-Edwards 2006 [18]	60 minutes (20 minutes of habituation)
Zhu et al. 2007 [19]	60 minutes (no habituation)
Li et al. 2009 [20]	3 hours (1 hour of habituation)
(Rivera-Quinones et al. 1998; McGavern et al. 1999 [1], [21]	3 days (no habituation)
Current Study	64 days (8 days of habituation)

## Materials and Methods

### Mice

SJL/J mice (Jackson Laboratories, Bar Harbor, ME) were housed and bred in Mayo Clinic's animal care facility. Animal protocols were approved by the Mayo Clinic Institutional Animal Care and Use Committee.

### Theiler's virus model of demyelination

Demyelinating disease was induced in 6- to 8-week-old female mice by intracerebral injection of TMEV. A 27-gauge needle delivered 10 µl containing 2.0×10^5^ plaque-forming units of Daniel's strain TMEV. This resulted in >98% incidence of infection with rare fatalities. All animals developed mild encephalitis, which resolved within 2 weeks. Animals worsen over the next 6–8 months with chronic spinal cord demyelinating disease. Axon injury and loss begin three months after infection correlating with neurologic dysfunction [3].

### Antibody Treatment

SJL mice (uninfected, 45 and 90 days post infection) were treated with a single 200 µg intraperitoneal dose of antibodies (rHIgM12 or isotype IgM control) dissolved in 0.5 ml of PBS. Third group was treated with 0.5 ml PBS only.

### Spontaneous activity monitoring

Spontaneous locomotor activity was recorded with Digiscan open field (OF) apparatus (Omnitech Electronics; Columbus, OH) and Versamax software, v.4.12-1AFE (Accuscan Instruments, Inc., Columbus, OH). The apparatus consists of six acrylic cages (40×40×30.5 cm) supported by a metal frame that holds two sets of photo cells. The device measures the number of discrete horizontal and vertical movements by tabulating the number of projected infrared beam interruptions. In all cages mice were exposed to identical environmental conditions: (a) freely accessible food and water, (b) a normal 12 h light/dark cycle, (c) 70°F ambient temperature. In each experiment hyperactive and, in rare occasion, overweight animals were excluded and the remainder of mice were randomized. Several groups of 5 SJL mice were placed in the center of the each cage and the baseline spontaneous activity was collected over the period of 8 consecutive days. After this period, three groups of mice with the most similar baseline activity were treated with rHIgM12, control IgM antibody or saline and then monitored over the period of 8 weeks. Data was collected as number of beam breaks per 1-hour blocks. The total horizontal and vertical activities were recorded using Versadat program, v.3.02-1AFE (Accuscan Instruments). We could not put more than 5 animals per activity box because this is the maximal number of animals allowed by Institutional Animal Care and Use Committee (IACUC).

### Data Analysis

Differences among treatment groups may be difficult to appreciate studying original unfiltered recordings (Figure 1A–C). To recover slow trends in the highly fluctuating original data we applied three different methods: 1) binning, 2) application of Gaussian low-pass filter (GF), and 3) polynomial fitting.

**Figure 2:**
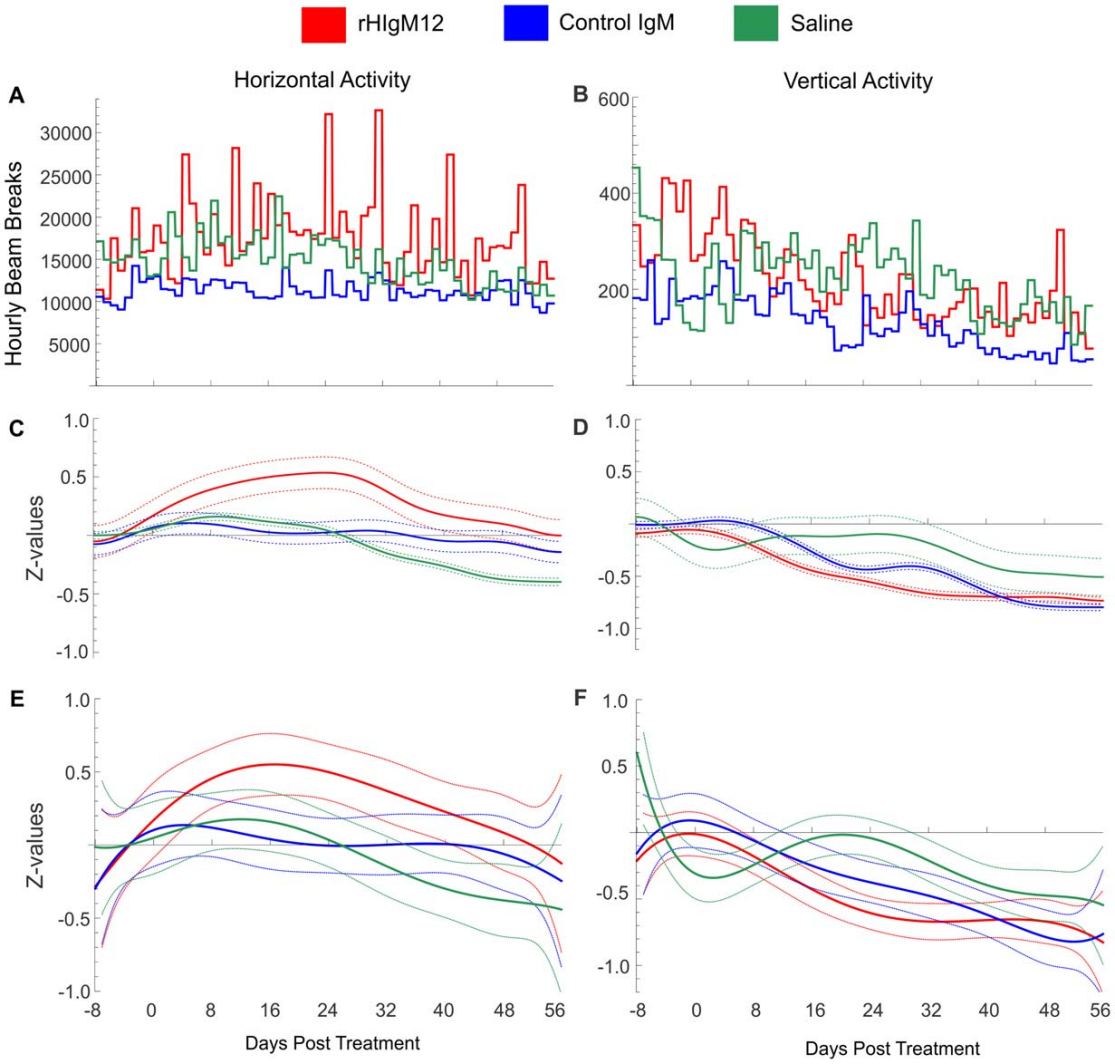
A single i.p. dose of neuron-binding antibody (rHIgM12) improves horizontal activity in chronically infected SJL mice. Three groups of 5 SJL mice at 90dpi were placed in AccuScan activity monitoring boxes. Baseline measurements were collected over 8 days. After the treatment mice were monitored continuously over 8 weeks. Panels A, C and E correspond to horizontal activity and B, D and F correspond to vertical activity. A, B) Average nocturnal activities presented as bins for each day suggested the improvement in horizontal activity only in rHIgM12-treated group; C) Gaussian filtering (GB = 2 days) and E) 6^th^ order polynomial fitting of standardized Z-values provided a clear and distinct improvement in rHIgM12-treated mice. B, D and F) Vertical activity was not affected by any treatment. Activity (vertical scale) is the number of beam interruptions per hour. Parameter GB can be interpreted as a value (in days) of the filter half width at the half height. With the increase of GB value the standard deviation decreases. The pointwise 95% confidence bands are given for each day.

Data binning is the simplest method and replaces a group of data points within a preselected bin by their average value. In our case, we selected a nocturnal bin given that murine activity peaks at night. Therefore, we replaced all nocturnal readings (12 h/day) by their average value as shown in Figures 2A, 2B, 4C, 4D, 6C and 6D. Comparison of groups can be performed by a simple *t*-test for a difference of means, with an effective sample size of 12 hours per treatment. Whereas these comparisons are simple, the small sample size at each time point results in large standard errors and statistical comparisons are of limited use. Overall, data binning is an effective method for visualizing noisy data, but is of limited use for statistical testing.

**Figure 3:**
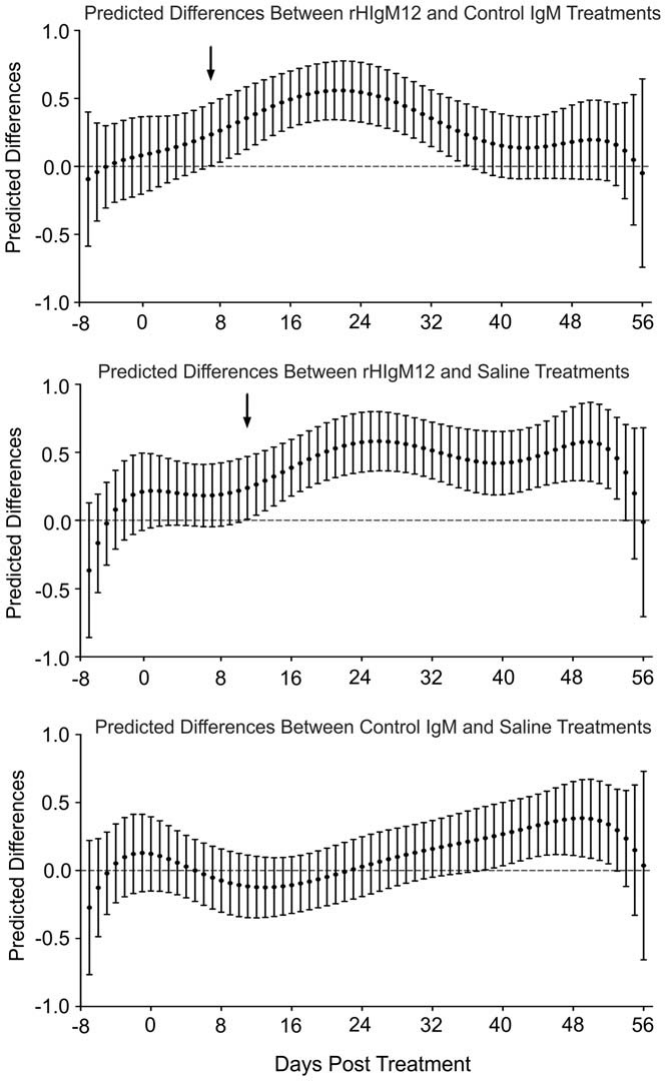
Comparison of three treatments at 90dpi by using predicted model values. Statistical analysis was performed for each day before and after the treatment. Horizontal nocturnal activity of rHIgM12-treated mice as compared to control IgM- and saline-treated mice significantly diverged/improved on day 7 (p = 0.045) and day 11 (p = 0.042) post-treatment, respectively (denoted by black arrows). The pointwise 95% confidence bands are given for each day.

**Figure 4:**
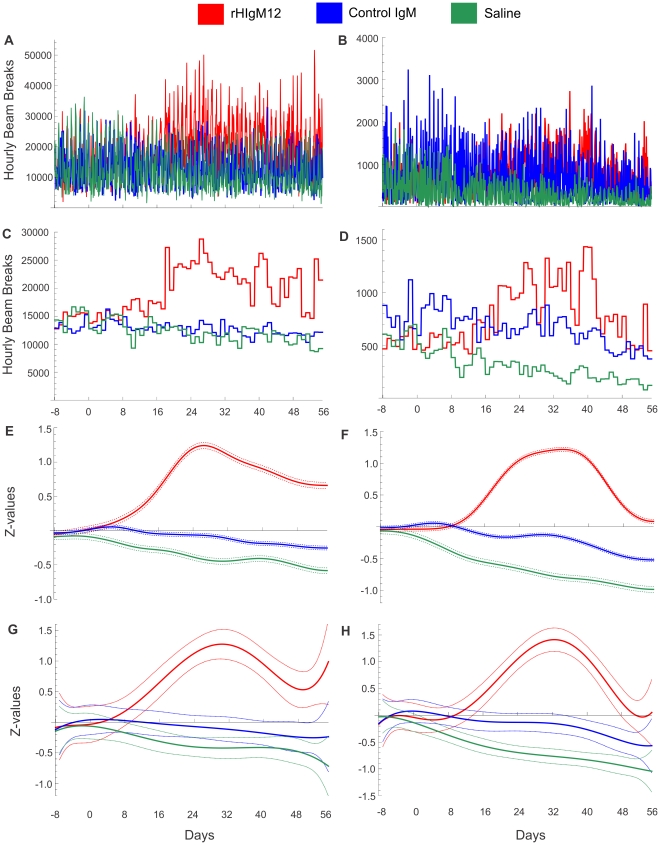
rHIgM12 improves both horizontal and vertical activity in SJL mice when given early in the disease. Three groups of 5 SJL mice at 45dpi were placed in AccuScan activity monitoring boxes. Baseline measurements were collected over 8 days. After the treatment mice were monitored continuously over 8 weeks. Panels A, C, E and G correspond to horizontal activity and B, D, F and H correspond to vertical activity. A, B) Original, unfiltered recordings for horizontal and vertical activity; C, D) Average nocturnal activities presented as bins for each day, E, F) Gaussian filtering (GB = 2 days) and G, H) 6^th^ order polynomial fitting of standardized Z-values revealed improvement in both horizontal and vertical activity in rHIgM12-treated group. Control IgM- and saline-treated groups showed similar levels of motor activity throughout the study. The pointwise 95% confidence bands are given for each day.

Gaussian filtering (GF) (also known as low-pass filtering, data smoothing, or sensitivity enhancement) is a noise-reduction procedure commonly used in Fourier transform spectroscopy and image processing [7]. GF, performed with an appropriate selection of Gaussian broadening (GB, in days) filtered out high-frequency fluctuations at a desired level. The filter choice is arbitrary and may be guided by the rate of expected activity changes. GF is a smoothing method using information from points taken from both sides of a value and weighting the influence of these points such that influence fades according to the Gaussian function. Computationally, GF can be implemented in two equivalent ways: 1) Fourier transformation (FT) of the data, followed by multiplication with a Gaussian function and inverse FT of the product; and 2) direct convolution of the data with a Gaussian kernel. In high-level software packages (Matlab (Mathworks) or Mathematica (Wolfram)) the Gaussian filter function is available with minimal or no programming by the user. With appropriate selection of GB the above detailed GF method enables simplified visualization of highly complex and heterogeneous data. One limitation of GF is that whereas it allows for easy visualization of trends, statistical comparisons are complicated by the choice of the GB.

For comparison and to demonstrate a method that allows for simplified statistical comparison, we fit polynomials to the data. These models allowed for polynomial terms up through the any degree (x^n^), and estimated shape parameters separately for each treatment group (interaction terms). Our choice of a 6^th^ degree polynomial was arbitrary after exploring several options, but allowed sufficient flexibility to model non-linear effects over time. Because the different treatment groups were optimally fit using varying order polynomials, we chose to allow for the higher order flexibility. Akaike Information Criteria (AIC) was used to determine the “best fit” of polynomial lines across several of the treatment groups [8]. AIC is a method of model comparison which balances the gain in R^2^ by adding additional terms, but penalizes for overcomplexity (i.e., use of degrees of freedom). In general, the 3^rd^ order fit was sufficient to capture the trend of the data, and for some cases the 2^nd^ order or even the linear fit was “best” according to AIC. However, our primary goal was to compare treatment groups at observed time points. Because of the large number of data points, and because we are not using the polynomial fit to predict (or extrapolate) treatment values outside of our observed data, the impact of “overfitting” on results is minimal.

One advantage of polynomial fitting is that the statistical comparison of treatment groups is straightforward: treatments can be compared at specified time points using the predicted model values and respective standard errors. Direct pairwise comparisons of treatments can be performed at regular intervals across the entire time frame to determine when treatment groups have significantly diverged. Finally, the polynomial fit removes additional medium and low frequency noise which focuses visual attention on the general trend over the entire time frame.

However, within each of the experiments some groups of mice had somewhat different baseline horizontal and vertical activity (8 days). Therefore, we first used Z-values to standardize baseline activity independently for each group and then performed Gaussian filtering or fitted polynomials to these values.

### Statistical analysis

Binning data and Gaussian low-pass filter smoothing of data were performed by using a macro written in Mathematica (Wolfram). Macro and instructions are available on http://mayoresearch.mayo.edu/mayo/research/rodriguez_lab/software.cfm. Polynomial regression models and statistical comparisons of treatment groups were performed using the predicted model values and respective standard errors based on the z-statistic (SAS Institute, Inc.). Direct pairwise comparisons of treatments were performed for each day across the entire time frame, and statistical significance was determined at the typical α = 0.05 threshold. No adjustments were made for multiple comparisons.

## Results

### rHIgM12 improves horizontal activity in SJL mice when given at 90dpi

Three groups of 5 SJL mice at 90 days post infection (dpi) were placed in activity boxes and baseline activity was measured for 8 consecutive days. Two groups of mice were treated with a single 200 µg dose of rHIgM12 or an isotype-control IgM. The third group was treated with saline. After the treatment, spontaneous activity was continuously recorded over 8 weeks. Because data was collected in 1-hour blocks, we obtained approximately 900 data points for each group. This original, raw data (Figure 1A-C) is highly fluctuating, so differences among treatment groups may be difficult to appreciate. All three methods (binning, gaussian filtering and polynomial fitting) revealed that mice treated with rHIgM12 showed improvement in horizontal activity, whereas control IgM- and saline-treated mice had no changes in the activity over the period of 8 weeks (Figure 2A, C and E). Using direct pairwise comparisons of three treatments after polynomial fitting, we determined that improvement in horizontal motor function in rHIgM12-treated mice became statistically significant at day 7 post treatment (as compared to control IgM) and at day 11 (as compared to saline) (Figure 3). Observed improvement in horizontal nocturnal activity of rHIgM12-treated animals persisted for approximately 30 days and then returned to baseline levels. Pairwise comparison of saline- versus control IgM-treatment groups showed statistical significance for days 38-52. However, when compared to major differences observed between rHIgM12-treatment group and the controls, we believe that these differences between the control groups are not biologically significant. On the other hand, vertical activity did not show major differences and was similar in all three groups (Figure 2B, D, and F).

**Figure 5:**
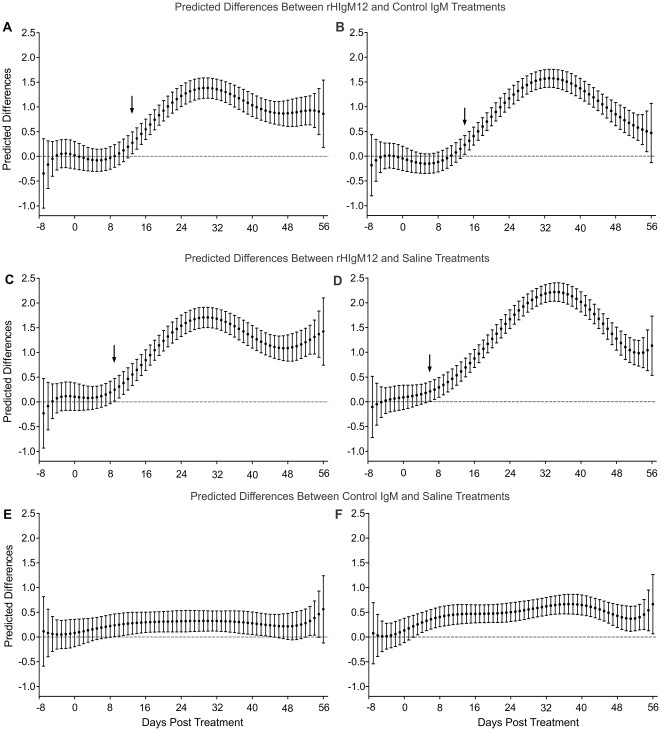
Comparison of three treatments at 45dpi by using predicted model values. Statistical analysis was performed for each day before and after the treatment. A, C) Horizontal nocturnal activity of rHIgM12-treated mice as compared to control IgM- and saline-treated mice significantly diverged on day 13 (p = 0.015) and day 9 (p = 0.036) post-treatment, respectively. B, D) Vertical (rearing) activity of rHIgM12-treated mice diverged/improved on day 14 (p = 0.019) and day 6 (p = 0.037) post-treatment, respectively. E, F) Comparisons of control IgM- vs. saline-treated mice did not reveal major biological changes neither for horizontal (E) nor for vertical (F) activity. Black arrows denote days with onset of significant improvement. The pointwise 95% confidence bands are given for each day.

### rHIgM12 improves horizontal and vertical activity in SJL mice when given at 45dpi

The previous study used vertical activity (rearing behavior) as a primary readout [1]. Due to axonal dropout in chronically TMEV-infected mice, hind limbs became progressively weak and stiff so rearing was reduced. In the first experiment of this study, when rHIgM12 was administered at the time of maximal demyelination (90dpi) and onset of progressive axonal loss, only horizontal activity was improved. The rearing behavior was not affected and was equivalent in all three treatment groups. Therefore we asked whether treatment at an earlier time point may be more beneficial. In a second experiment, groups of 5 mice were treated at 45 dpi with a single 200 µg dose of rHIgM12, an isotype-control IgM or saline. An identical experimental design was used; baseline activity was collected for 8 days, and post-treatment activity for 8 weeks. In this experiment, beginning around 2 weeks post-treatment, rHIgM12-treated mice showed clear improvements in both horizontal and vertical activity. This was evident after using all three methods: averaging data, applying Gaussian filter or polynomial fitting (Figure 4C–H). Control IgM- and saline-treated mice showed similar activity levels throughout the end of study. In rHIgM12-treated mice, improvement in horizontal activity was evident until the experiment ended. On the other hand, improvement in vertical activity lasted for about 4 weeks and then dropped to baseline values. Direct pairwise comparisons of three treatments showed that improvement in horizontal motor function in rHIgM12-treated mice significantly diverged at day 13 post treatment (as compared to control IgM) and at day 9 (as compared to saline) (Figure 5A and C). Likewise, vertical motor function in rHIgM12-treated mice significantly diverged at day 14 post treatment (as compared to control IgM) and at day 6 (as compared to saline) (Figure 5B and D). Comparison of control IgM- versus saline-treated groups did not reveal major biological differences for either horizontal or vertical activity (Figure 5E and F).

**Figure 6:**
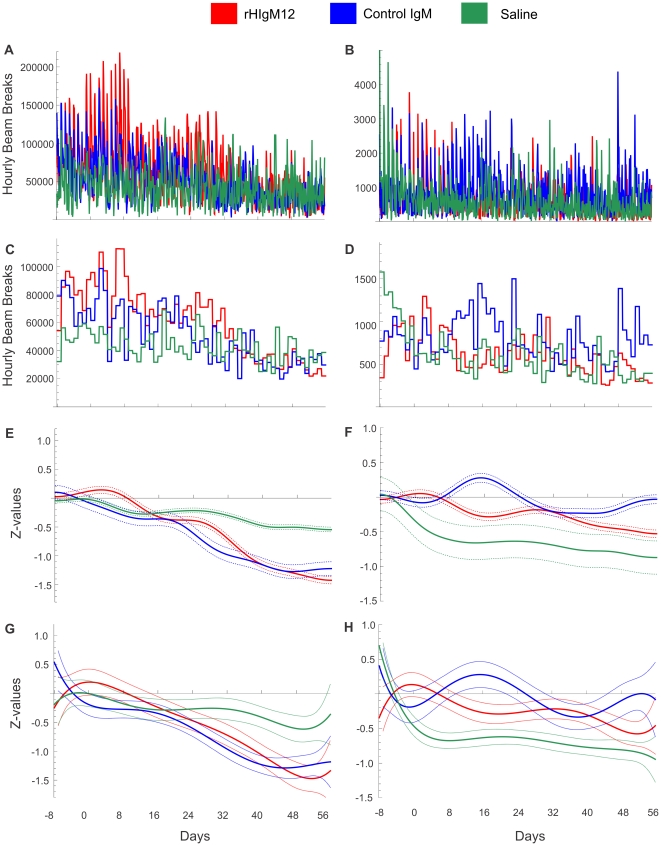
Normal, uninfected SJL mice have highly variable spontaneous activity which is not influenced by any treatment. Three groups of 5 uninfected SJL mice were placed in AccuScan activity monitoring boxes. Baseline measurements were collected over 8 days. After the treatment mice were monitored continuously over 8 weeks. None of the treatments induced changes in either horizontal (A, C, E and G) or vertical (B, D, F and H) activity. The pointwise 95% confidence bands are given for each day.

### rHIgM12 does not alter the spontaneous activity in normal uninfected mice

Treatment with rHIgM12 in previous two experiments showed clear beneficial effect in neurologically disabled infected mice. To exclude the possibility that this antibody may have irritable properties and therefore elicits increased motor function, we performed similar experiment with uninfected mice. Three groups of age matched uninfected mice were treated with rHIgM12, control IgM or saline. Compared to the activity enhancement in infected mice, rHIgM12 as well as the other two treatments did not induce any increase in motor function of normal mice (Figure 6). All three groups showed a tendency to decline in spontaneous activity. This result indicates that none of the antibodies has an influence on increasing activity in normal mice.

## Discussion

There is a critical need for the development of neuroprotective therapies for multiple sclerosis as well as for other demyelinating and neurodegenerative diseases. Even though there are some anti-inflammatory drugs that may indirectly lead to a decrease in axonal damage during inflammatory CNS disease, there are no drugs that act directly at the level of neurons/axons. The main goal of neuroprotection is to limit neuronal dysfunction and to attempt to maintain the functional integrity of neurons and axons. For many years demyelination, the pathological hallmark of MS was considered as a cause of permanent neurological deficits. It is clear now that demyelination is necessary, but not sufficient for permanent axonal loss [9]. Demyelination only predisposes denuded axons to the secondary injury caused by either T-cell cytotoxicity or loss of local neurotrophic support from dead oligodendrocytes [10].

Previous observation that neuron-binding antibody sHIgM12 promoted robust neurite extension [5] represents a clear beneficial *in vitro* response. Because recombinant version of this antibody showed similar in vitro properties we asked whether it would affect motor activity of mice with TMEV-induced demyelinating disease. Analysis of motor function was performed by monitoring the spontaneous nocturnal activity. First, we treated mice at the time of maximal demyelination and onset of axonal loss (90dpi). Eight weeks after the treatment, rHIgM12 improved only horizontal motor activity, whereas vertical activity was not affected. However, when mice were treated earlier in the disease (at 45dpi), rHIgM12 improved both horizontal and vertical activity. In the chronic TMEV-induced disease, rearing behavior (vertical activity) is most severely affected and it appears that degeneration and loss of axons responsible for this activity is irreversible. Conversely, the early phase of the disease, when these axons are not irreversibly injured, appears to be ideal time for the treatment. Jones et al. utilized EAE model and by studying axonal dropout and motor function they proposed an identical paradigm; treatment with neuroprotective drugs should begin early in the disease, even before the onset of motor deficits [11]. Second, because the functional improvement occurs about two weeks after the treatment and starts to fade approximately 25–30 days later, it may be that repeated treatments will be necessary to sustain motor function. Unfortunately in our studies, it was not possible to test multiple doses of human IgMs because of anti-human antibody immune response in mice which results in anaphylaxis. A2B5 is a mouse monoclonal antibody that also promotes neurite outgrowth [5] and represents a likely candidate to test single versus multiple dosing on functional outcome and its duration of action. Finally, none of the treatments had an effect on motor function of uninfected, normal animals. Irrespective of the treatment all groups of normal mice showed a gradual decline in spontaneous activity, which may be explained by habituation to the environment. This decline of activity in normal animals makes rHIgM12-induced increase in activity of diseased mice even more impressive.

We have demonstrated improvement in motor function in a chronic progressive model of inflammatory demyelinating disease in which has been generally very difficult to prevent the development of neurologic deficits. Therefore, neuron-binding monoclonal antibody rHIgM12 represents very promising therapeutic agent for the treatment not only of human MS, but possibly also other demyelinating or neurodegenerative disorders. In addition, because there are examples of clinically silent MS attacks [12], [13], we and others have proposed that neuroprotective compounds should be complemented with immunomodulatory agents [11]. We propose that the combined treatment of immunomodulatory drugs and rHIgM12 may result in a significant enhancement of CNS preservation and repair following axonal injury.

Taken together, results from this study provide three important conclusions: 1) the stage of the disease when the treatment is given is critical (early treatment is more beneficial); 2) to further maintain improved motor function, there may be a need for repeated treatments and 3) rHIgM12 is not toxic and does not affect motor function in normal, uninfected animals. With five mice per cage, we cannot rule out the possibility that our findings were due to chance. The highly statistically significant differences between the groups argue that the results are not due to chance. Our findings were consistent across multiple experiments with mice from different time points post infection. In addition, mice were randomly chosen for inclusion and all had the same genetic background. The findings are consistent with the hypothesis that a recombinant antibody that targets neurons improve neurologic function in a chronic axonal model of demyelination.
